# Correction

**DOI:** 10.1080/0886022X.2024.2356372

**Published:** 2024-06-03

**Article title:** Development and validation of a LASSO-based prediction model for immunosuppressive medication nonadherence in kidney transplant recipients

**Authors:** Dong, L., Zhu, X., Zhao, H., et al.

**Journal:** *Renal Failure*

**Bibliometrics:** Volume 45, Number 02, 2238832

**DOI:** https://doi.org/10.1080/0886022X.2023.2238832

When the article was first published online, Table 2 was differently presented compared to its original scale.

**Table 2.** Adherence to IM measured by BAASIS.

Item number		*N* (%)
1A	Taking non-adherence: Yes/No	218 (21.6%)/793 (78.4%)
	1 time	166 (16.4%)
	2 or more times	52 (5.2%)
1B	Drug-holidays: Yes/No	122 (12.1%)/889 (87.9%)
	1 time	94 (9.3%)
	2 or more times	28 (2.8%)
2	Timing adherence: Yes/No	281 (27.8%)/730 (72.2%)
	1 time	151 (14.9%)
	2–3 times	98 (9.7%)
	4–5 times	15 (1.5%)
	Every 2–3 days	14 (1.4%)
	Almost every day	3 (0.3%)
3	Dose-alteration: Yes/No	62 (6.2%)/949 (93.8%)
4	Discontinuation: Yes/No	33 (3.3%)/978 (96.7%)

Table 2 should be formatted as per below in accordance to the scale used, Basel Assessment of Adherence to Immunosuppressive Medications Scale (BAASIS).

